# The development and validation of the Research for Practice Tool (R4PT) for nursing and midwifery

**DOI:** 10.1186/s12913-025-13112-x

**Published:** 2025-09-30

**Authors:** Se Ok Ohr, Vicki Parker, Michelle Giles, Sophie Dilworth, Jean Ball, Ashleigh Stuart, Madeleine Hinwood, Maralyn Foureur, Gena Lieschke

**Affiliations:** 1 Nursing and Midwifery Research Centre, Hunter New England Local Health District, Gate Cottage, Building 6, James Fletcher Campus, 72 Watt Street, Newcastle, NSW 2300 Australia; 2 School of Nursing and Midwifery, College of Health, Medicine and Wellbeing, University of Newcastle, University Drive, Callaghan, NSW 2308 Australia; 3 Hunter Medical Research Institute, 1 Kookaburra Close, New Lambton Heights, NSW 2305 Australia; 4 School of Medicine and Public Health, College of Health, Medicine, and Wellbeing, University of Newcastle, University Drive, Callaghan, NSW 2308 Australia

**Keywords:** Research and development, Research capacity, Research impact, Research questionnaire, Nursing, Midwifery, Delphi study

## Abstract

**Background:**

Tools for assessing research capacity and participation for health professionals have been in use for over a decade with little change. Given the evolving research context emphasising integration into clinical practice, it is time to update or develop tools that reflect current research practices. The aim of this study was to develop and validate a questionnaire designed to examine nurses’ and midwives’ attitudes, capabilities, participation, and perceived impact of practice-based research.

**Methods:**

The Research for Practice Tool (R4PT) was developed using factors and items identified from an extensive literature review and analysis of existing tools. A modified Delphi method was used to confirm the factors and items. The content validity of the R4PT was determined through reviews from four research experts and six nursing and midwifery clinician researchers. The usability and acceptability of the R4PT was conducted by 12 nurses and midwives. The factors and items of the R4PT were assessed by factor analyses of responses from a target population of 8500 nurses and midwives in a Local Health District in NSW, Australia.

**Results:**

A total of 1,430 participants responded to the R4PT (17%). A seven-factor solution was identified in the exploratory factor analysis. Sixty-six out of 73 items each loaded onto a single factor, explaining 71.5% of the variance. The identified factors were research value and culture (1 and 2), research integration and relevance for practice, research translation, research impact, individual research capability and team research capability. The factors were distinct with the inter-factor correlations less than 0.8. Confirmatory factor analysis indicated that all models showed good fit, with non-significant chi-squared tests, CFIs of 1, TLIs > 0.95, RMSEAs of 0 and SRMRs < 0.8. Cronbach’s alpha for all factors, except research value and culture 1, showed acceptable consistency (> 0.7).

**Conclusions:**

The R4PT is a valid and reliable means of assessing research participation, aligning with clinical practice and service delivery trends. The culture and value factors (1 and 2) need to be reworked and retested prior to inclusion in subsequent surveys. The R4PT will provide valuable information to inform capacity-building activities, workforce and work practice models that integrate research into practice.

**Supplementary Information:**

The online version contains supplementary material available at 10.1186/s12913-025-13112-x.

## Background

There is well-documented evidence that supports the benefits of research-engaged organisations. Benefits cited include better patient outcomes, cost savings and better staff satisfaction [1, 2]. The place of research in clinical practice has changed significantly over the last decade, with a mandated shift to research that links to clinical practice with tangible outcomes and impact [2, 3]. Commentators, funding bodies and researchers alike agree that research without a connection to purpose and outcomes is a lost opportunity to improve the human experience and economic value. Focus on the translation of research has seen the development of staged pathways that create connections from knowledge creation through application, escalation and evaluation [3]. This development has enhanced the potential for research to have meaning and relevance for end-users and the participation of stakeholders as co-researchers. Translational research links stakeholders, their concerns and the processes that shift to greater satisfaction, effectiveness, and efficiency. At the same time demystification, normalisation, and domestication of research through education and exposure potentially places it within the realm of clinicians and their clinical practice environment.

The role of researchers and their relationship with practice and consumers of research has been called into question, with many strategic developments aiming to better connect researchers and practice, and to encourage the participation of clinicians. However, tools devised to measure health professionals’ research engagement remain unchanged. A review of existing tools and their capacity to reflect the changing research in practice landscape is necessary to support research participation and build research capacity.

There are numerous studies of research capacity and culture amongst health professionals reported in the literature. However, they are limited by small sample sizes, and most pertain to allied health professionals [4–8]. These previous examinations of research capacity among health professionals in health care contexts have primarily focused on research skills, support for research at various levels, and research culture and value [9, 10]. Reviews of nurses’ and midwives’ participation in research conducted over the last two decades have tended to focus on barriers to participation, consistently reporting a lack of knowledge, time, and support as impediments [11–13]. Historically, nurses and midwives have had an uncomfortable relationship with research which has been described as unreasonable, unrealistic, and separate to the work of clinicians [12, 14, 15].

In recent years, the narrative has started to change with research participation being increasingly supported and encouraged. There is now a significant and growing body of nursing and midwifery research and evidence of an emerging more positive view of research and its importance. For example, in a qualitative study by Scala et al, [16] in the USA, nurses described being involved in research as empowering, legitimising the profession, and impacting the future. It also reported that research participation was seen as an opportunity to learn and grow professionally and contribute to achieving the best outcomes.

An Australian study conducted by the authors in 2016 [1, 15] examined nurses’ and midwives’ research capacity, activity, and interest across the Local Health District (LHD) (*n* = 721, response rate = 10%), identified that the nature and extent of research participation was variable across sites, individuals and clinical specialties. In many cases, interest and involvement in research was not sustained. Participants identified the need for greater incentives and structural support. Most important was the need for research to have tangible meaning for patients and clinical practice [1].

In Australia, changes in government policy arising in relation to health research have highlighted the imperative for research to have outcomes and impact [17]. This change has enabled expansion and resourcing of research conducted within practice contexts with clinicians and consumers. There is a growing need and opportunity to build research capacity amongst nurses and midwives and to lead research on priority clinical issues.

What we don’t understand is the degree to which nurses and midwives are now involved, the nature of the roles they play, and the nature and scope of impacts on nurses and midwives, their practice and their professional goals and aspirations. Our assumption is that there is no longer an expectation that nurses and midwives conduct and be responsible for research as an individual researcher alone, but instead function as a member of a transdisciplinary research team, ideally with the knowledge and support of their colleagues and organisation.

Considering these changes, there is a need to re-evaluate concepts and tools used to inform understanding of the participation of clinicians in practice-based research now and into the future.

## Methods

### Aim

To develop and validate a questionnaire to investigate nurses’ and midwives’ attitudes toward research participation in practice-based research, together with perceptions of impact.

### Design

This study was conducted using a 3-phase design. Phase 1 included a comprehensive review of the literature sourcing concepts, trends and existing tools was conducted. Phase 2 used a modified Delphi method to develop, and validate the questionnaire called the ‘Research for Practice Tool’ (R4PT). Phase 3 was the final distribution R4PT cross-sectional online survey of nurses and midwives.

#### Phase 1: development of the research for practice tool (R4PT)

A review of empirical and grey literature was carried out to identify concepts, theories, models, frameworks, and trends pertaining to practice-based research from the last 10 years (2014–2024), together with tools used to measure research participation. Five electronic data bases (CINHAL, Medline, Embase, Proquest, Web of Science) were searched using comprehensive search terms that included research, nurses and midwives, capacity, and involvement (see Supplementary material 1). Grey literature was sourced from Local Health District, State and Federal Government sites and agencies, and research institutes. Included studies were limited to English only. Two authors independently abstracted and thematically grouped the extracted data.

Overall, there is increasing focus on translation of research, research outcomes and increasing application of theories and frameworks to inform research practice to achieve these goals. The review identified five key dimensions. These were (i) research capacity and capability, (ii) research culture and support, (iii) relevance and integration, (iv) research translation and (v) impact. Available tools were evaluated according to inclusion of each of the dimensions. Research capacity and capability (individual, team and organisational) were identified as fundamental elements of research participation and hence the most surveyed [1, 18–20]. Having supportive culture and valuing of research were deemed to be critical to research participation and integration in practice. Support was seen to be both multifaceted and multi-levelled, coming from within the immediate practice environment but also from health service executive, managers, and the education sector. In the studies we reviewed this level of support was largely absent. For example, organizations did not always support high interest levels and commitment to research at the individual level [5, 6]. Research training, time to undertake research, and opportunities were not equally allocated among professional groups [21, 22]. Much literature also pointed out that the lack of support impacted nursing and midwifery research, with limited financial and human resources, and a perceived lack of value and culture for research [1, 5, 6, 18, 23–25].

Within the context complexity of health care and fast-changing medical technologies, research integration and relevance to practice, were emphasised to ensure positive patient and practice outcomes [2, 20]. Integration of research into usual practice closes the gap between research and clinical practice. It requires that research be based in practice and practice-led, involving collection and interrogation of nuanced local data, along with dedicated dialogue (through meetings and other communicative processes) and resources. In this way, research is dynamic and goal-directed and requires the involvement of consumers, along with clinicians, across all stages of the research process.

Translation of research focuses on its implementation, evaluation, and ability to achieve sustainable outcomes. Clinician researchers bring practice-based knowledge and the capacity and connections to ensure research has meaning and is translated to practice in a timely fashion [2, 26, 27]. Utilisation of implementation approaches, such as knowledge translation and implementation science, build in support through engagement of key stakeholders, including managers, clinicians, and consumers [28–31]. Translation necessarily involves teams and teamwork. Nurses and midwives, as front-line workers, are a necessary part of many implementation research projects and are critical to success.

Research impact is how research has tangible meaning, value, and purpose for individuals and society. Knowledge, innovation, health, and quality of life for individuals, populations, and societies, improved consumer and clinician experience and satisfaction and economic benefits were recognized as research impact [2, 27]. Active and continuous experiential learning within research partnerships in individuals, organizations, and health and social care systems with policy and context was emphasized as research impact [2, 16, 22, 27]. For example, the Australian Council of Research insists that a research proposal should include its ability to have tangible benefits for patients, families, communities, populations, and health services. This could enhance efficiencies of utilisation and allocation of resources, to improve practice, reduce waste, reduce negative environmental consequences, and to improve access to services as well as to address inequity and disadvantage in health care. Further, some authors highlight the benefits for clinicians in terms of professional development or career opportunities in nursing and midwifery [5, 6, 18, 32].

The review and analysis of existing literature identified seven tools measuring various aspects of research capacity, culture, and participation at individual, team, and organizational levels [8, 15, 33–37]. The Research Capacity and Culture (RCC) tool was the most frequently used and focused on research capacity, culture, and support [10]. While the RCC was developed for allied health professionals [8], over fifty studies used it to measure research capacity development, and seven of these studies included nurses or midwives [5–7, 15, 38–40]. However, only a small number of nurses and midwives participated. The Research and Development Culture Index (R & D Culture Index) assesses the strength of an organization’s research and development culture by capturing the role of the individual practitioner and the organizational environment [33]. The Nursing Research Questionnaire (NRQ) focuses on Nurses’ research capability, research-related activity, and factors influencing research development [34]. The Nursing Research Self-Efficacy Scale (NURSES) measures individual nurse’s degree of research self-efficacy and their perceptions regarding their unit’s collective support of research [35]. The Hunter New England Local Health District Nursing & Midwifery Research Survey (HNELHD NMS) assesses research activity, capacity, capability, and culture, as well as previously identified barriers and enablers to research activity practice and participation [15]. This tool also includes questions about research translation. The Wessex Research Network (WReN) Spider [36] measures the research experience of health care practitioners, using a bibliographic search. Additional questions look at wider issues of research success, including collaborators, resources, and environment. VICTOR (making Visible the ImpaCT Of Research) assesses the impact of delivering research within an organization. It focuses on the potential impacts related to changes in workforce, skills and knowledge, service delivery, patient and carer experience, economic benefits, and changes in culture [37]. VICTOR was used to report the impact of specific projects.

Existing tools were evaluated for their attention to each of the five dimensions of research identified in the integrative review, as shown in Table 1. Whilst the previously adopted concepts of culture and capacity used in the existing tools remain relevant, they were limited in their ability to assess research integration into practice, research translation and impact in the current context of clinical practice.

**Table 1:** Mapping of the previous tools

	**Research capabilities and expertise **	**Research culture and value **	**Research integration and relevance for practice**	**Research translation**	**Research impact**
RCC	Included	Included	Included		
R & D Culture Index	Included	Included	Included		
NRQ	Included	Included			
NURSES	included	Included	Included		
HNELHD NMS	Included	Included	Included	Included	
WReN SPIDER	Included				Included
VICTOR	Included	Included		Included	Included

Based on the mapping of tools and the findings of the literature review, we developed the R4PT. The R4PT uses the key constructs that reflect changes in the research environment.

#### Phase 2: the modified Delphi

**Fig. 1:**
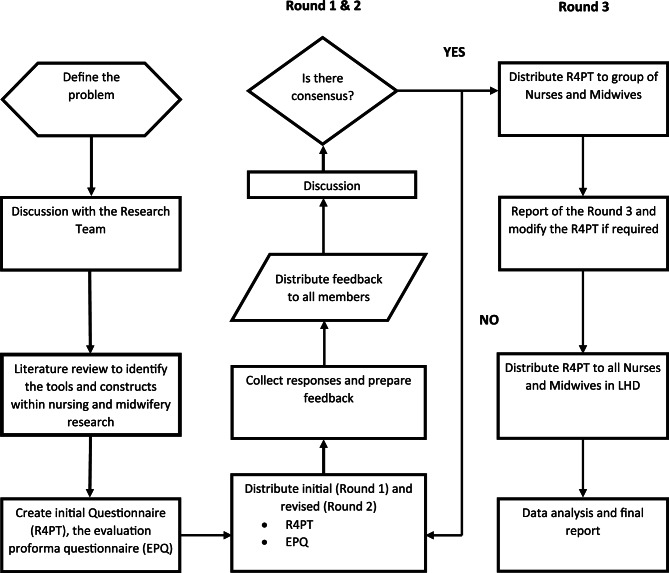
Delphi processes.

A modified Delphi approach was used (see Fig. 1) to establish consensus among a group of expert nurses and midwives with experience conducting practice-based research [41]. The Delphi process consisted of three rounds to obtain expert opinion and consensus on a suite of indicators and measures developed by the research team.

##### Round one: identifying key concepts and trends of the nursing and midwifery research

Four research experts (two nurse researchers, one midwife researcher and one statistician) were provided with the literature review results, the derived concepts and working definitions developed for each of the proposed constructs and the proposed R4PT. The experts were asked to review the overall tool, each construct, and corresponding items and to comment on relevance, significance, comprehensiveness, clarity, duplication, bias, and utility and asked to make suggestions for improvement if and where necessary using an evaluation proforma (See Supplementary material 2). Individual experts sent their independent responses to the facilitators and the summary of the responses was discussed in a collaborative meeting to reach consensus. The results of this exercise were used to refine the R4PT for distribution to a larger group of clinician researchers.

##### Round two: seeking consensus on the draft R4PT

Six nursing and midwifery clinician researchers from two LHDs and two universities in two different states of Australia were recruited to further refine the constructs of the R4PT. The facilitators sent blind carbon copy emails to these researchers to assess construct validity and concurrent validity with several important outcomes of nursing and midwifery research. Also, interrater agreement between experts’ opinions, and feedback as an additional parameter of reliability was assessed. Four responses from individual experts (66%) were aggregated and a consensus to modify the R4PT was reached. Further modifications were made after Round Two: several measures were updated to increase clarity, and a question was added to Sect. 5 (“Provide an example: Describe how a research project you have been involved with came about.”).

##### Round three: testing the usability and acceptability of the R4PT

Twelve nurses and midwives in different role classifications were asked to complete the R4PT via REDCap electronic data capture tools hosted at Hunter New England LHD [42, 43] to evaluate relevance to nursing and midwifery research in practice, ease of access and use, acceptable time spent for completion and further suggestions were requested. Eight respondents (66%) reported that the R4PT was easy to understand and relevant to Nursing and Midwifery research, and completion of the R4PT was easy. Some modifications were made to clarify confusion between the terms ‘clinical team’ and ‘research team’. Time to complete the R4PT was reported as 10–20 min.

#### The final indicators and measures for R4PT

**Fig. 2:**
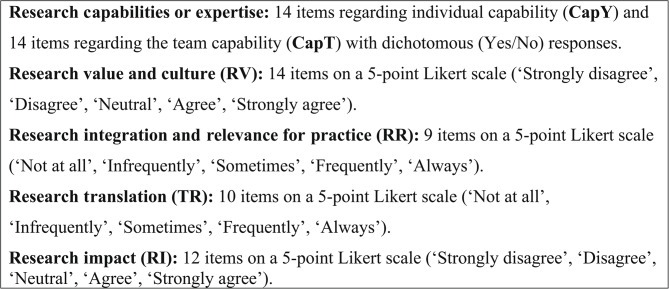
Components of the original R4PT

The final indicators and measures were established after three rounds of the Delphi technique (See Fig. 2). Demographic items were also included: gender, age, employment, length of employment, classification of position, location of role, research-related post-graduate studies, research education, and training.

#### Phase 3: final R4PT distribution

The R4PT was distributed to all nurses and midwives (approximately 8,500) of a large LHD, in New South Wales, Australia. The LHD encompasses a major metropolitan centre, and regional communities, with a small percentage of people located in remote communities.

### Data collection

Data were collected using an online survey via REDCap [42, 43]. An invitation to complete the survey was sent to the entire population of nurses and midwives in the LHD via their organisational email address and a text message to their mobile contact number listed with the LHD. An information statement was included with the email/text and was included as the front page to the online survey. The information explained that by completing the survey participants were consenting to participate in the research. A reminder email/text was sent to all invitees a fortnight after the first email/text. The survey was open for 12 weeks (May to August 2023).

### Statistical analysis

Analyses were conducted in R version 4.3.1 [44]. Due to the large amount of missing data, multiple imputation was performed using the R package ‘mice’ [45] using fully conditional specification. Fifty imputations were generated using proportional odds models and logistic regression models including variables hypothesised to be associated with the scales and those correlated with the variable to be imputed. Each imputation was randomly split, with half used for exploratory factor analysis (EFA) and half for confirmatory factor analysis (CFA).

#### Exploratory factor analysis (EFA)

The EFA was conducted using the ‘psych’ package [46]. As the measured variables were binary and ordinal, polychoric and tetrachoric correlations were used. Appropriateness was tested using Bartlett’s test of sphericity and the Kaiser-Meyer-Olkin (KMO) test of factorial adequacy. A statistically significant Bartlett’s test of sphericity (*p* < 0.05) and a KMO statistic greater than 0.5 were used to ensure the correlation matrix was appropriate for EFA [47].

Scree plots, parallel analysis and Velicer’s MAP were employed to find the ideal number of factors. Solutions ranging from 6 factors (the expected number of factors based on survey design) to 13 factors were examined with iterated principal axis extraction, as the data was categorical [47], and ‘oblimin’ rotation, as factors were expected to be correlated.

To retain a variable, it had to laod onto a single factor with a pattern loading factor of 0.4 or greater and have communality of at least 0.4 [48]. To retain a factor, it must have been loaded by at least 3 variables. Based on methods by Norman and Streiner [49], a statistically significant pattern loading factor at the 1% level was determined to be 0.19. As this was unlikely to be clinically significant, salience was defined as a pattern loading factor greater than 0.4.

#### Confirmatory factor analysis (CFA)

For the CFA, two models were tested, one with six factors and one with seven factors. The first had variables loading onto factors based on the original survey design (the expected model). The second had factors loaded per the EFA. For each model, the scales of the latent variables were fixed by fixing the factor loading of the first indicator to 1 and all latent variables were allowed to be correlated.

Pooled chi-squared statistics, comparative fit indices (CFI), root mean square error of approximations (RMSEA) and standardised root mean squared residuals (SRMR) are shown along with the range of Tucker Lewis Indexes (TLI) from each imputed dataset for each model. Ideally models that fit the data have a non-significant chi-square goodness of fit test, CFI > 0.95, TLI > 0.95, RMSEA < 0.06 and SRMR < 0.08 [50]. However, when comparing models, simply a higher CFI and TLI and lower RMSEA and SRMR (close to 0) were used to indicate a better fit. Parameter estimates with 95% confidence intervals are shown for the model with factors loading as in the EFA.

#### Factor descriptive statistics and internal consistency

Response frequencies and descriptive statistics were based on non-imputed data, while reliability, measured using Cronbach’s alpha, was calculated from the imputed data. An overall Cronbach alpha greater than 0.7 was considered reasonable and greater than 0.8 was very good. However, scores greater than 0.9, may suggest multicollinearity.

## Results

### Demographics

A total of 1,430 participants responded to the R4PT (17% response rate). There was 11.36% missing data overall, with 50.56% complete cases. Of the scale variables, research impact had 42.9% missing, translational research had 40.9% missing, research integration and relevance for practice had 36.0-36.4% missing and research value and culture had 27.4 to 28.5% missing.

90% of respondents were female and 45% were employed full-time. 13% of respondents were aged < 30 years, 45% were aged 30–49 years, and 42% were aged 50 years or over. 55% were employed as registered nurses or midwives. There was a variety of experience, with a majority (60%) greater than 3 years. 44% practiced in a metropolitan area, 49% in a rural area and 7% worked in both areas. 2% had obtained or were currently undertaking a Doctor of Philosophy (PhD), 2% had completed or were currently completing a Masters by research, and 22% had completed or were currently completing a Masters by coursework.

### Exploratory factor analysis (EFA)

The inter-item polychoric and tetrachoric correlations varied from less than 0.001 to 0.963. Bartlett’s test of sphericity and the KMO measure of sampling adequacy objectively showed the variables were sufficiently correlated and the sample was adequate to justify factor analysis, with a p-value < 0.05 (< 0.001) and a KMO > 0.5 (0.75).

A seven-factor solution emerged as most appropriate, as models with additional factors failed to achieve the minimum criterion of three variables loading onto each factor. Factors emerged mostly as expected, however, the research value and culture variables measured 2 separate factors. Two items (RV_SuppMgr and RV_ClinPrac) were dropped as they did not load onto any factors (See Supplementary material 3. Table S1). CapY_Econ loaded onto 2 factors, and CapY_Abor, RV_Relevant, Rv_Supp_Oth and RV_Oppor had communality below 0.4 and were sequentially removed. After rotation, this solution explained 71.5% of the variance, as seen in Table 2. The inter-factor correlations for each factor are distinct, as correlations are less than 0.8 (see Supplementary material 3. Table S2).

**Table 2:** Pattern matrix of 7-factor EFA solution

Variable	Items	Factor 1	Factor 2	Factor 3	Factor 4	Factor 5	Factor 6	Factor 7	Communality	Uniqueness
RV_CritRole	1. N&M play a critical role in research	0.03	0.08	0.07	0.02	-0.01	0.16	**0.64**	0.53	0.47
RV_Contrib	3. N&M can make an important contribution to a research team	-0.01	0.03	0.08	-0.05	0.02	0.07	**0.76**	0.62	0.38
RV_Everyday	4. Research is part of everyday practice in our unit	0.07	0.02	0.01	-0.01	0.10	**0.60**	0.10	0.46	0.54
RV_Worth	5. Research is worth doing	0.01	-0.11	-0.02	0.02	0.10	0.01	**0.78**	0.63	0.37
RV_Valued	7. Research is openly valued as part of core business	0.02	0.03	-0.07	0.15	-0.09	**0.60**	0.11	0.46	0.54
RV_IndivPlan	8. Research is evident in my individual role expectation and appraisal	0.08	0.01	0.00	0.01	0.10	**0.61**	0.04	0.46	0.54
RV_ServPlan	9. Research is evident in our Service strategic plan	-0.01	0.02	0.02	0.08	0.05	**0.74**	0.00	0.64	0.36
RV_Recogn	10. There is increasing recognition of importance of research in our unit	0.08	0.07	-0.02	0.13	0.06	**0.62**	0.09	0.61	0.39
RV_Benefit	14. Research experience is beneficial to career progression of N&M	0.08	0.15	-0.02	-0.06	0.04	0.01	**0.60**	0.43	0.57
RR_ClinPrac	1. Relevant to clinical practice	-0.07	-0.04	0.09	0.19	**0.57**	0.13	0.11	0.61	0.39
RR_Clinician	2. Derived and developed by clinicians	0.08	0.07	0.03	0.11	**0.62**	0.04	0.03	0.61	0.39
RR_Patient	3. Informed by patient experiences, preferences, and satisfaction	0.05	0.06	-0.09	0.02	**0.72**	0.10	-0.01	0.66	0.34
RR_QualProj	4. Derived from a quality project	0.00	-0.03	-0.02	0.13	**0.73**	0.04	-0.03	0.67	0.33
RR_Intervent	5. Related to an intervention (eg. drug, procedure, treatment)	0.06	-0.04	-0.02	0.16	**0.72**	-0.07	-0.03	0.63	0.37
RR_Moc	6. Related to a model of care	-0.01	0.09	0.01	-0.06	**0.82**	0.00	0.00	0.68	0.32
RR_ClinProb	7. Designed to solve a clinical problem	-0.04	0.07	0.06	0.02	**0.81**	-0.01	0.04	0.75	0.25
RR_ExistData	8. Derived from or uses existing clinical data	-0.04	0.01	0.00	-0.03	**0.85**	0.01	0.07	0.74	0.26
RR_ChgePrac	9. Used to inform changes and practice in our unit	0.02	0.06	-0.05	0.09	**0.74**	0.04	0.02	0.75	0.25
TR_Interdisc	1. Research activities are interdisciplinary in nature	0.05	0.01	0.09	**0.63**	0.11	0.08	-0.02	0.58	0.42
TR_ReschClin	2. Research activities are done collaboratively between researchers and clinicians	0.07	-0.03	0.03	**0.72**	0.13	0.08	-0.10	0.67	0.33
TR_Patients	3. Research activities are done in collaboration with recipients of care	0.02	0.01	-0.01	**0.75**	0.10	0.09	-0.04	0.74	0.26
TR_Priorities	4. Research activities are directed by service strategic priorities	0.00	-0.02	0.03	**0.78**	0.09	0.04	-0.10	0.68	0.32
TR_PttOutcs	5. Research findings have improved patient, client and community outcomes	-0.01	0.15	0.06	**0.70**	0.04	-0.02	0.08	0.73	0.27
TR_OrgOutcs	6. Research findings have improved organisational outcomes	-0.04	0.26	0.03	**0.66**	0.04	-0.02	0.05	0.78	0.22
TR_PrcChg	7. Research findings have resulted in sustainable practice change	-0.03	0.18	-0.02	**0.72**	0.03	0.02	0.06	0.78	0.22
TR_Support	8. Research activities are well supported by management	0.02	0.05	-0.12	**0.70**	0.04	0.12	0.02	0.68	0.32
TR_Impact	9. Research is used to evaluate the impact of interventions on patient outcomes	-0.02	0.11	0.00	**0.72**	0.06	0.03	0.07	0.76	0.24
TR_Consumers	10. Consumer/ community contributions are valued in research.	0.03	0.12	-0.03	**0.68**	0.05	-0.01	0.08	0.67	0.33
RI_ClinChg	1. Clinical practice change	0.00	**0.62**	-0.02	0.16	0.11	0.00	0.07	0.68	0.32
RI_Policy	2. Policy and guideline change	0.01	**0.64**	-0.04	0.12	0.09	-0.02	0.03	0.63	0.37
RI_NewKnow	3. New knowledge and innovation	-0.01	**0.73**	0.02	0.10	0.08	-0.01	0.09	0.75	0.25
RI_Efficient	4. Enhanced efficiency and streamlined care coordination	0.03	**0.78**	0.02	0.08	0.08	0.02	0.00	0.79	0.21
RI_CloseGap	5. Closing the Gap	0.08	**0.74**	-0.13	0.06	-0.05	0.01	-0.04	0.61	0.39
RI_Economics	6. Economic benefits	-0.02	**0.76**	0.05	0.18	-0.12	0.03	-0.06	0.67	0.33
RI_PtOutcm	7. Patient outcomes	-0.05	**0.84**	0.02	0.04	0.05	0.01	0.08	0.83	0.17
RI_SocOutcm	8. Social and community benefits	0.02	**0.87**	-0.04	-0.05	0.03	0.05	-0.03	0.75	0.25
RI_PtExp	9. Patient experience and satisfaction	-0.04	**0.85**	0.01	-0.10	0.09	0.00	0.08	0.74	0.26
RI_Staff	10. Staff experience and satisfaction	0.01	**0.85**	-0.01	0.04	-0.05	0.01	-0.01	0.74	0.26
RI_QualSafe	11. Quality and safety	0.02	**0.80**	-0.02	0.03	0.13	-0.04	0.01	0.78	0.22
RI_Enviro	12. Reduction of negative environmental impact	-0.02	**0.75**	-0.02	0.04	-0.04	0.07	-0.15	0.60	0.40
CapY_Prop	1. Designing research and developing a research proposal– Individual	-0.10	-0.02	**0.88**	0.08	-0.12	-0.04	0.18	0.80	0.20
CapT_Prop	Team	**0.93**	-0.02	0.04	0.02	-0.01	0.07	-0.04	0.90	0.10
CapY_Litsch	2.Undertaking literature search- Individual	0.19	0.02	**0.76**	-0.02	0.09	-0.15	0.03	0.76	0.24
CapT_Litsch	Team	**0.87**	0.07	0.04	0.06	-0.02	0.01	0.02	0.81	0.19
CapY_Fund	3. Applying for funding- Individual	0.05	-0.16	**0.69**	0.11	0.00	-0.05	-0.02	0.53	0.47
CapT_Fund	Team	**0.90**	0.08	0.07	-0.04	0.00	-0.01	0.05	0.88	0.12
CapY_Consum	4.Engaging consumers- Individual	0.32	-0.01	**0.57**	-0.02	0.01	-0.13	-0.01	0.58	0.42
CapT_Consum	Team	**0.77**	0.00	0.07	0.09	0.04	-0.14	0.00	0.66	0.34
CapT_Abor	5.Assessing impact on Indigenous people and communities– Team	**0.85**	0.11	0.14	-0.03	0.00	-0.01	-0.08	0.84	0.16
CapY_Stakeh	6.Engaging non-consumer stakeholders (clinicians, managers, policymakers)- Individual	0.22	0.05	**0.70**	0.02	-0.10	-0.08	0.13	0.70	0.30
CapT_Stakeh	Team	**0.89**	0.04	0.06	0.02	0.04	-0.08	-0.02	0.84	0.16
CapY_Ethics	7. Applying for ethics approval -Individual	0.02	-0.04	**0.82**	0.06	-0.10	-0.02	0.08	0.72	0.28
CapT_Ethics	Team	**0.93**	0.02	0.00	-0.06	0.02	0.07	0.00	0.88	0.12
CapY_Data	8. Collecting data- Individual	0.21	0.07	**0.83**	-0.14	0.06	0.03	-0.06	0.85	0.15
CapT_Data	Team	**0.88**	-0.06	-0.17	0.11	-0.01	-0.12	0.07	0.70	0.30
CapY_Datman	9. Managing data- Individual	0.06	0.05	**0.90**	-0.07	-0.01	0.15	-0.11	0.84	0.16
CapT_Datman	Team	**0.97**	-0.06	-0.14	0.06	0.05	-0.05	0.06	0.87	0.13
CapY_Stat	10.Conducting statistical analysis- Individual	-0.11	-0.04	**0.84**	-0.04	0.10	0.04	-0.10	0.64	0.36
CapT_Stat	Team	**0.95**	0.02	0.02	-0.10	0.00	0.04	0.00	0.92	0.08
CapY_Qual	11.Analysing qualitative data- Individual	-0.16	-0.04	**0.89**	-0.06	0.08	0.03	0.03	0.73	0.27
CapT_Qual	Team	**0.97**	-0.06	-0.04	0.01	-0.01	0.06	-0.01	0.92	0.08
CapT_Econ	12. Conducting an economic evaluation- Individual	**0.91**	0.04	0.07	-0.07	-0.04	0.08	-0.01	0.90	0.10
CapY_Report	13. Writing up of research findings- Individual	0.05	-0.08	**0.84**	0.10	0.00	-0.02	0.01	0.76	0.24
CapT_Report	Team	**0.97**	-0.05	0.00	0.03	-0.01	0.02	0.00	0.94	0.06
CapY_Public	14. Preparing and submitting findings for publication- Individual	0.01	0.03	**0.86**	0.05	-0.03	-0.01	0.05	0.75	0.25
CapT_Public	Team	**0.94**	-0.05	0.08	0.00	-0.02	0.06	0.02	0.94	0.06

Proportion of variance accounted for = 0.715

Boldface indicates where pattern loading criteria were met, i.e. where variables loaded onto a factor with a pattern loading factor of 0.4

###  Confirmatory factor analysis

CFA compared the fit of the EFA model, to the expected model. Fit statistics are shown in Table 3. All models show good fit, with non-significant chi-squared tests, CFIs of 1, TLIs > 0.95, RMSEAs of 0 and SRMRs < 0.8, however the model with 7 factors was slightly better fitting, with a lower SRMR and higher TLIs.

**Table 3:** Fit Indices

Model	chi-squared (df)	*p*-value^1^	CFI^2^	RMSEA^3^	SRMR^4^	TLI^5^
Expected	1029.62 (2330)	1	1	0	0.07	0.994 - 0.994
EFA	287.41 (2123)	1	1	0	0.05	0.998 - 0.999

^1^chi-squared goodness of fit test

^2^Comparative Fit Index

^3^Tucker-Lewis Index; Range of values from imputed datasets

^4^Root mean square error of approximation

^5^Standardised Root Mean Squared Residual

### CFA factor loadings

The seven identified factors were: research value and culture 1, research value and culture 2, research integration and relevance for practice, research translation, research impact, individual research capability and team research capability. Factors were distinct as correlations were less than 0.8 (See Supplementary material 3. Table S3).

Standardised factor loadings from the CFA model with indicators loading factors per the EFA can be seen in Table 4. All variables statistically significantly load on the latent variables.

**Table 4:** Factor loadings from CFA

Factor	Variable	Standardised estimate	Estimate (95% CI)	p-value
Research value and culture - 1	RV_CritRole	0.75	1 (1, 1)	
	RV_Contrib	0.79	1.07 (0.9, 1.23)	<0.001
	RV_Worth	0.81	1.08 (0.91, 1.25)	<0.001
	RV_Benefit	0.69	0.93 (0.76, 1.1)	<0.001
Research value and culture - 2	RV_Everyday	0.67	1 (1, 1)	
	RV_Valued	0.66	0.99 (0.82, 1.15)	<0.001
	RV_IndivPlan	0.65	0.96 (0.82, 1.11)	<0.001
	RV_ServPlan	0.80	1.19 (1.01, 1.36)	<0.001
	RV_Recogn	0.84	1.25 (1.06, 1.43)	<0.001
Research integration and relevance for practice	RR_ClinPrac	0.77	1 (1, 1)	
	RR_Clinician	0.79	1.03 (0.96, 1.11)	<0.001
	RR_Patient	0.79	1.03 (0.96, 1.1)	<0.001
	RR_QualProj	0.80	1.04 (0.97, 1.12)	<0.001
	RR_Intervent	0.77	1 (0.93, 1.07)	<0.001
	RR_Moc	0.80	1.04 (0.97, 1.11)	<0.001
	RR_ClinProb	0.85	1.1 (1.03, 1.18)	<0.001
	RR_ExistData	0.82	1.07 (1, 1.14)	<0.001
	RR_ChgePrac	0.89	1.16 (1.09, 1.23)	<0.001
Research translation	TR_Interdisc	0.78	1 (1, 1)	
	TR_ReschClin	0.82	1.06 (1.01, 1.11)	<0.001
	TR_Patients	0.82	1.06 (1, 1.11)	<0.001
	TR_Priorities	0.80	1.03 (0.98, 1.09)	<0.001
	TR_PttOutcs	0.87	1.12 (1.07, 1.18)	<0.001
	TR_OrgOutcs	0.89	1.15 (1.1, 1.21)	<0.001
	TR_PrcChg	0.88	1.13 (1.08, 1.19)	<0.001
	TR_Support	0.78	1 (0.93, 1.07)	<0.001
	TR_Impact	0.88	1.14 (1.08, 1.19)	<0.001
	TR_Consumers	0.84	1.08 (1.02, 1.14)	<0.001
Research impact	RI_ClinChg	0.86	1 (1, 1)	
	RI_Policy	0.81	0.95 (0.91, 0.99)	<0.001
	RI_NewKnow	0.86	1.01 (0.97, 1.04)	<0.001
	RI_Efficient	0.86	1 (0.97, 1.03)	<0.001
	RI_CloseGap	0.73	0.85 (0.79, 0.9)	<0.001
	RI_Economics	0.77	0.9 (0.85, 0.94)	<0.001
	RI_PtOutcm	0.88	1.02 (0.99, 1.06)	<0.001
	RI_SocOutcm	0.83	0.97 (0.93, 1.01)	<0.001
	RI_PtExp	0.86	1.01 (0.97, 1.04)	<0.001
	RI_Staff	0.82	0.96 (0.92, 1)	<0.001
	RI_QualSafe	0.87	1.01 (0.97, 1.04)	<0.001
	RI_Enviro	0.71	0.83 (0.78, 0.88)	<0.001
Individual research capability	CapY_Consum	0.85	1 (1, 1)	
	CapY_Data	0.94	1.1 (0.99, 1.22)	<0.001
	CapY_Datman	0.93	1.09 (0.99, 1.2)	<0.001
	CapY_Ethics	0.84	0.99 (0.86, 1.12)	<0.001
	CapY_Fund	0.69	0.81 (0.65, 0.97)	<0.001
	CapY_Litsch	0.88	1.03 (0.92, 1.14)	<0.001
	CapY_Prop	0.88	1.03 (0.92, 1.15)	<0.001
	CapY_Public	0.93	1.09 (0.98, 1.2)	<0.001
	CapY_Qual	0.91	1.06 (0.96, 1.17)	<0.001
	CapY_Report	0.93	1.09 (0.99, 1.2)	<0.001
	CapY_Stakeh	0.83	0.97 (0.85, 1.09)	<0.001
	CapY_Stat	0.83	0.97 (0.85, 1.1)	<0.001
Team research capability	CapT_Abor	0.91	1 (1, 1)	
	CapT_Consum	0.89	0.98 (0.92, 1.04)	<0.001
	CapT_Data	0.89	0.97 (0.91, 1.03)	<0.001
	CapT_Datman	0.96	1.05 (1, 1.1)	<0.001
	CapT_Econ	0.98	1.08 (1.03, 1.12)	<0.001
	CapT_Ethics	0.98	1.07 (1.02, 1.12)	<0.001
	CapT_Fund	0.96	1.05 (1, 1.1)	<0.001
	CapT_Litsch	0.88	0.96 (0.89, 1.03)	<0.001
	CapT_Prop	0.96	1.05 (1, 1.1)	<0.001
	CapT_Public	0.99	1.08 (1.04, 1.13)	<0.001
	CapT_Qual	0.99	1.08 (1.04, 1.13)	<0.001
	CapT_Report	0.98	1.08 (1.03, 1.12)	<0.001
	CapT_Stakeh	0.95	1.04 (1, 1.09)	<0.001
	CapT_Stat	0.99	1.08 (1.03, 1.13)	<0.001

### Factor descriptive statistics and internal consistency

Descriptive statistics, including response frequencies, means, standard deviations, medians, minimums and maximums are shown for each variable by factor in Supplementary material 3. Tables S4-S10. In addition, these tables show Cronbach’s alpha, a measure of internal consistency and correlation of the item with the scale. Both the correlation of the item with the entire scale and correlation of the item with the scale without the variable in it are also shown, the higher these measures are, the greater the indicator is correlated with the factor. All factors showed acceptable consistency (> 0.7) except research value and culture 1. Team research capability and research translation both had very high alphas (0.97 and 0.92 respectively) which suggests possible collinearity.

## Discussion

As researchers with responsibility for supporting nurses and midwives to undertake research in practice contexts, we sought to design a tool that would align with the changing research landscape. This evolving landscape is characterised by increasing opportunity, developing availability and sophistication of resources such as access to targeted funding and to statistical and economic expertise. We are acutely aware of the inequitable availability and distribution of these resources amongst health professionals, with nurses and midwives under-resourced and underrepresented. This inequity is amplified in rural contexts. We are aware that nurses and midwives are members of interdisciplinary research teams and there is a need to understand and enhance the roles they play in these teams. We required a tool that would help us understand and engage effectively with this emerging landscape and to monitor improvement and impact. Building on previous studies we used a rigorous process to identify measures that represent changing directions in practice-based research that would tell us about the nature and participation of nurses and midwives in research, together with changing perceptions of research.

The literature review and the Delphi study enabled a consensus from nursing and midwifery research experts and nurses and midwives on a core set of relevant and feasible measures for research conducted in practice contexts by and with nurses and midwives. Although our review included a search for both nursing and midwifery research, most studies were done by and pertained to nursing only. Midwife researchers and midwives were included in the Delphi panel.

The R4PT reflects practice-based research, focusing on relevance and integration into practice and reflecting the strong relationship between nursing and midwifery research and the perceived impacts and value of research for practice. Research integration items indicate ownership of research and research outcomes, emphasising translation and focusing on engagement with and participation in a research team. Skills and resources identified as necessary to conduct research in the current context are included, emphasizing the engagement of consumers and stakeholders, economic evaluations, and implementation research activities.

The evidence for the construct validity of the R4PT was further supported by exploratory and confirmatory factor analyses. In this study, EFA of the R4PT yielded a seven-factor model that explained 71.5% of the variance. CFA was used to validate the EFA derived factor structures of the R4PT, the results indicated good fit for the R4PT, offering confirmatory evidence for the factor structure. All factors of the R4PT, as measured by Cronbach’s alpha, were good, with consistency greater than 0.8 except research value and culture 1 (0.69) and 2 (0.75). Research values and culture was the only scale that contained negatively worded variables. Each of these were removed as they did not load or had low communality, however changing the phrasing of these may be enough to improve their correlation. The research values and culture 1 and 2 items will be reworked and retested before inclusion in subsequent surveys. Although the value and culture items did not load on a single factor, these concepts feature strongly in other items that focus on integration, teamwork and availability of specific supports, i.e. relevance and integration, translation and impact. Team research capability had extremely high consistency with many variables with inter-item correlations above 0.9. As such, CapT_Data and CapT_Datman will be combined. CapT_Report and CapT_Public will also be combined, along with the corresponding Individual research capability variables (CapY_Data/CapY_Datman and CapY_Report/CapY_Public). The revised R4PT will include demographics and five factors: Research capabilities (12 items individual and 12 items team); Research integration and relevance for practice (9 items); Research Translation (10 items); Research Impact (12 items).

### Limitations and recommendations

This study represents an initial step in the development and validation of the R4PT. Some limitations are to be considered. The response rate was calculated with an assumption that all 8500 nurses and midwives received an invitation to participate in the R4PT but it was unknown how many emails were actually read. Although the response rate was low, the number of responses for all items was adequate for reliable analysis. The rate of completion of R4PT dropped for items at the end of the tool such as research impact (43%), translational research (41%) and integration and relevance for practice (almost 36%). This could indicate respondent fatigue or difficulty responding to the items. EFA identified that the wording of some items may have been problematic, as all the negatively worded items had low communality. In recognition of these limitations, the R4PT has been modified.

## Conclusion

To the knowledge of the authors, this is the first study to both develop and undertake a detailed validation of a questionnaire to assess the items and measure contemporary nursing and midwifery research in practice. This study indicates that the R4PT is a valid and reliable instrument for assessing nursing and midwifery research in practice. The R4PT can be used to assess nurses’ and midwives’ participation in research. The successful impact of their participation is contingent on researchers, policy makers and health managers having a shared understanding of the key drivers and processes of research implementation and the ways in which research can be embedded as an integral part of clinical practice. This R4PT also provides valuable information that informs capacity building activities as well as workforce and work practice models that integrate research in practice. With fast-changing health contexts and research environments, the R4PT will assist nursing and midwifery research to go forward to improve quality of care and patient safety. The R4PT will need further testing with nurses and midwives in other contexts and a wider range of health professionals.

## Supplementary Information

Supplementary Material 1.

Supplementary Material 2.

Supplementary Material 3.

## Data Availability

The datasets used and/or analysed during the current study are available from the corresponding author on reasonable request.

## Abbreviations

CFA: Confirmatory Factor Analysis
CFI: Comparative Fit Indices
EFA: Exploratory Factor Analysis
KMO: Kaiser-Meyer-Olkin
LHD: Local Health District
R4PT: Research for Practice Tool
RMSEA: Root Mean Square Error of Approximations
SRMR: Standardised Root Mean Squared Residuals
TLI: Tucker Lewis Indexes
