# Supplementary material for: The development and validation of the Research for Practice Tool (R4PT) for nursing and midwifery
Source: BMC Health Serv Res. 2025 Sep 30;25:1245. doi: 10.1186/s12913-025-13112-x (PMC12482206; doi:10.1186/s12913-025-13112-x)
Supplement: Supplementary file 1 — Supplementary Material 1. [file 12913_2025_13112_MOESM1_ESM.docx]

**Research for Practice Tool Review**

Thank you for taking the time to review the questionnaire. Can you answer the following questions in reviewing the tool each of the inter-related components?

1. **Is the aim of the questionnaire clear and unambiguous for respondents?**

- Yes
- No

Comment

-----------------------------------------------------------------------------------------------------------------------------------------------------------------------------------------------------------------------------------------------------------------------------------------------------------------------------------------------------------------------------------------------------------------

1. **How well does the questionnaire address its intended aim?**

- not at all
- in part
- in full

1. **Is the questionnaire well-constructed in terms of progression of constructs and questions?**

- Yes
- No

Comment

------------------------------------------------------------------------------------------------------------------------------------------------------------------------------------------------------------------------------------------------------------------------------------------------------------------------------------------------------------------------------------------------------------------

1. **The questionnaire is easy to read and understand?**

- Yes
- No

Comment:

---------------------------------------------------------------------------------------------------------------------------------------------------------------------------------------------------------------------------------------------------------------------------------------------------------------------------------------------------------------------------------

1. **Are all constructs meaningful for the purpose of the questionnaire?**

|  | Yes | No | Comment |
| --- | --- | --- | --- |
| Research capabilities and expertise |  |  |  |
| Research culture and value |  |  |  |
| Research Integration and relevance for practice |  |  |  |
| Research translation |  |  |  |
| Research impact |  |  |  |

**5a. Is there anything important missing? If so please suggest what else needs to be included.**

**____________________________________________________________________________________________________________________________________________________________________________________________________________________________________________________________________________________________________________**

1. **Are all items in each section clearly related and reflect an aspect of the construct being measured?**

|  | Yes | No | Comment |
| --- | --- | --- | --- |
| Research capabilities and expertise |  |  |  |
| Research culture and value |  |  |  |
| Research Integration and relevance for practice |  |  |  |
| Research translation |  |  |  |
| Research impact |  |  |  |

1. **Is what is being asked of respondents reasonable and not onerous?**

- Yes
- No

Comment:_____________________________________________________________________________________________________________________________________________________________________________________________________________________________________________

1. **Is there enough opportunity to gather additional information through open ended questions?**

- Yes
- No

Comment where and what extra information would be useful.

________________________________________________________________________________________________________________________________________________________________________________________________________________________________________________________________________________________________________________________________________

**Any other comments**

**____________________________________________________________________________________________________________________________________________________________________________________________________________________________________________________________________________________________________________________________________________________________________________________________________________________________________________________________________________________________________________**

**Thank you.**
