# Supplementary material for: The development and validation of the Research for Practice Tool (R4PT) for nursing and midwifery
Source: BMC Health Serv Res. 2025 Sep 30;25:1245. doi: 10.1186/s12913-025-13112-x (PMC12482206; doi:10.1186/s12913-025-13112-x)
Supplement: Supplementary file 2 — Supplementary Material 2. [file 12913_2025_13112_MOESM2_ESM.docx]

**Additional file 3.**

**The development and validation of a research for practice tool for Nursing and Midwifery**

**Table S1: Items dropped from the analysis**

| **Factor** | **Variable** | **Items** |
| --- | --- | --- |
| Factor 4 Your individual research capacity | CapY_Abor  CapY_Econ | 5. Assessing impact for Aboriginal people and communities  12. Conducting an economic evaluation |
| Factor 6 Research value and culture | RV_Relevant  RV_ClinPrac  RV_Oppor  RV_SuppMgr  Rv_Supp_Oth | 2. Research is a specialist activity that is relevant to only a few N&M  6. In our unit, research creates more problems than it is worth  11. Opportunities to learn to do research are readily available to N&M  12. My manager is reluctant to support staff to participate in research  13. In our unit, N&M support each other to conduct research |

**Table S2: EFA Inter-factor correlation**

| Factor 1 | Factor 2 | Factor 3 | Factor 4 | Factor 5 | Factor 6 | Factor 7 |
| --- | --- | --- | --- | --- | --- | --- |
| 1.00 | 0.08 | 0.38 | 0.08 | 0.07 | 0.08 | 0.08 |
| 0.08 | 1.00 | -0.07 | 0.65 | 0.52 | 0.32 | 0.18 |
| 0.38 | -0.07 | 1.00 | -0.04 | 0.00 | -0.02 | 0.16 |
| 0.08 | 0.65 | -0.04 | 1.00 | 0.67 | 0.33 | 0.19 |
| 0.07 | 0.52 | 0.00 | 0.67 | 1.00 | 0.35 | 0.25 |
| 0.08 | 0.32 | -0.02 | 0.33 | 0.35 | 1.00 | 0.17 |
| 0.08 | 0.18 | 0.16 | 0.19 | 0.25 | 0.17 | 1.00 |

**Table S3: CFA Inter-factor correlation**

| Factor 1 | Factor 2 | Standardised estimate |
| --- | --- | --- |
| Research value and culture 1 | Research value and culture 2 | 0.46 |
|  | Research impact | 0.26 |
|  | Individual research capability | 0.34 |
|  | Team research capability | 0.19 |
| Research value and culture 2 | Research impact | 0.53 |
|  | Individual research capability | 0.04 |
|  | Team research capability | 0.22 |
| Research integration and relevance for practice | Research impact | 0.68 |
|  | Individual research capability | 0.04 |
|  | Team research capability | 0.25 |
| Research translation | Research impact | 0.79 |
|  | Individual research capability | 0.06 |
|  | Team research capability | 0.23 |
| Research impact | Individual research capability | 0.03 |
|  | Team research capability | 0.18 |
| Individual research capability | Team research capability | 0.60 |

**Table S4: Research value and culture – 1:** response frequencies, descriptive statistics, reliability and correlation

|  | Response Frequencies | | | | | | | Descriptive Statistics | | | | | Alpha | | Correlation | |
| --- | --- | --- | --- | --- | --- | --- | --- | --- | --- | --- | --- | --- | --- | --- | --- | --- |
| Variable | 1 | 2 | 3 | 4 | 5 | Missing | n | Mean | SD | Median | Min. | Max. | Raw | Std | Scale^a^ | Drop^b^ |
| RV_CritRole | 0.01 | 0.02 | 0.10 | 0.41 | 0.47 | 0.27 | 1,038 | 4.29 | 0.81 | 4 | 1 | 5 | 0.60 | 0.62 | 0.58 | 0.47 |
| RV_Contrib | 0.01 | 0.01 | 0.05 | 0.43 | 0.51 | 0.28 | 1,034 | 4.43 | 0.67 | 5 | 1 | 5 | 0.59 | 0.60 | 0.61 | 0.50 |
| RV_Worth | 0.01 | 0.00 | 0.08 | 0.41 | 0.49 | 0.28 | 1,032 | 4.37 | 0.74 | 4 | 1 | 5 | 0.60 | 0.61 | 0.59 | 0.49 |
| RV_Benefit | 0.02 | 0.05 | 0.22 | 0.45 | 0.27 | 0.29 | 1,022 | 3.90 | 0.91 | 4 | 1 | 5 | 0.66 | 0.66 | 0.49 | 0.41 |
| Overall | | | | | | | | 16.85^c^ | 2.33 |  | | | 0.68 | 0.69 | 0.35 |  |
| ^a^Correlation of the item with the scale, adjusted for item overlap and scale reliability | | | | | | | | | | | | | | | | |
| ^b^Correlation of the item with the scale without the item in it | | | | | | | | | | | | | | | | |
| ^c^Mean of sum of items | | | | | | | | | | | | | | | | |

**Table S5: Research value and culture – 2:** response frequencies, descriptive statistics, reliability and correlation

|  | Response Frequencies | | | | | | | Descriptive Statistics | | | | | Alpha | | Corr | |
| --- | --- | --- | --- | --- | --- | --- | --- | --- | --- | --- | --- | --- | --- | --- | --- | --- |
| Variable | 1 | 2 | 3 | 4 | 5 | Missing | n | Mean | SD | Median | Min. | Max. | Raw | Std | Scale^a^ | Drop^b^ |
| RV_Everyday | 0.10 | 0.21 | 0.26 | 0.27 | 0.16 | 0.28 | 1,034 | 3.18 | 1.23 | 3 | 1 | 5 | 0.72 | 0.72 | 0.55 | 0.48 |
| RV_Valued | 0.07 | 0.15 | 0.38 | 0.29 | 0.11 | 0.28 | 1,029 | 3.21 | 1.04 | 3 | 1 | 5 | 0.71 | 0.72 | 0.55 | 0.48 |
| RV_IndivPlan | 0.08 | 0.20 | 0.38 | 0.26 | 0.08 | 0.28 | 1,028 | 3.07 | 1.05 | 3 | 1 | 5 | 0.71 | 0.71 | 0.57 | 0.50 |
| RV_ServPlan | 0.05 | 0.16 | 0.39 | 0.31 | 0.09 | 0.28 | 1,027 | 3.21 | 0.99 | 3 | 1 | 5 | 0.69 | 0.69 | 0.65 | 0.56 |
| RV_Recogn | 0.07 | 0.19 | 0.33 | 0.31 | 0.10 | 0.28 | 1,026 | 3.20 | 1.07 | 3 | 1 | 5 | 0.69 | 0.69 | 0.65 | 0.55 |
| Overall | | | | | | | | 15.83^c^ | 3.82 |  | | | 0.75 | 0.75 | 0.38 |  |
| ^a^Correlation of the item with the scale, adjusted for item overlap and scale reliability | | | | | | | | | | | | | | | | |
| ^b^Correlation of the item with the scale without the item in it | | | | | | | | | | | | | | | | |
| ^c^Mean of sum of items | | | | | | | | | | | | | | | | |

**Table S6: Research integration and relevance for practice:** response frequencies, descriptive statistics, reliability and correlation

|  | Response Frequencies | | | | | | | Descriptive Statistics | | | | | Alpha | | Correlation | |
| --- | --- | --- | --- | --- | --- | --- | --- | --- | --- | --- | --- | --- | --- | --- | --- | --- |
| Variable | 1 | 2 | 3 | 4 | 5 | Missing | n | Mean | SD | Median | Min. | Max. | Raw | Std | Scale^a^ | Drop^b^ |
| RR_ClinPrac | 0.07 | 0.09 | 0.27 | 0.31 | 0.27 | 0.36 | 915 | 3.62 | 1.17 | 4 | 1 | 5 | 0.87 | 0.87 | 0.64 | 0.60 |
| RR_Clinician | 0.10 | 0.14 | 0.36 | 0.28 | 0.13 | 0.36 | 913 | 3.20 | 1.13 | 3 | 1 | 5 | 0.87 | 0.87 | 0.65 | 0.61 |
| RR_Patient | 0.09 | 0.14 | 0.38 | 0.29 | 0.11 | 0.36 | 912 | 3.19 | 1.08 | 3 | 1 | 5 | 0.87 | 0.87 | 0.65 | 0.61 |
| RR_QualProj | 0.09 | 0.11 | 0.41 | 0.29 | 0.09 | 0.36 | 911 | 3.18 | 1.06 | 3 | 1 | 5 | 0.87 | 0.87 | 0.67 | 0.63 |
| RR_Intervent | 0.10 | 0.09 | 0.35 | 0.34 | 0.11 | 0.36 | 911 | 3.26 | 1.10 | 3 | 1 | 5 | 0.87 | 0.87 | 0.64 | 0.60 |
| RR_Moc | 0.08 | 0.12 | 0.35 | 0.34 | 0.11 | 0.36 | 910 | 3.26 | 1.08 | 3 | 1 | 5 | 0.87 | 0.87 | 0.66 | 0.62 |
| RR_ClinProb | 0.09 | 0.12 | 0.33 | 0.36 | 0.11 | 0.36 | 910 | 3.29 | 1.08 | 3 | 1 | 5 | 0.87 | 0.87 | 0.72 | 0.67 |
| RR_ExistData | 0.08 | 0.11 | 0.39 | 0.33 | 0.09 | 0.36 | 910 | 3.25 | 1.03 | 3 | 1 | 5 | 0.87 | 0.87 | 0.69 | 0.65 |
| RR_ChgePrac | 0.09 | 0.10 | 0.32 | 0.35 | 0.14 | 0.36 | 910 | 3.36 | 1.11 | 3 | 1 | 5 | 0.87 | 0.87 | 0.72 | 0.68 |
| Overall | | | | | | | | 29.55^c^ | 7.13 |  | | | 0.88 | 0.88 | 0.46 |  |
| ^a^Correlation of the item with the scale, adjusted for item overlap and scale reliability | | | | | | | | | | | | | | | | |
| ^b^Correlation of the item with the scale without the item in it | | | | | | | | | | | | | | | | |
| ^c^Mean of sum of items | | | | | | | | | | | | | | | | |

**Table S7: Research translation:** response frequencies, descriptive statistics, reliability and correlation

|  | Response Frequencies | | | | | | | Descriptive Statistics | | | | | Alpha | | Correlation | |
| --- | --- | --- | --- | --- | --- | --- | --- | --- | --- | --- | --- | --- | --- | --- | --- | --- |
| Variable | 1 | 2 | 3 | 4 | 5 | Missing | n | Mean | SD | Median | Min. | Max. | Raw | Std | Scale^a^ | Drop^b^ |
| TR_Interdisc | 0.12 | 0.14 | 0.38 | 0.28 | 0.08 | 0.41 | 848 | 3.05 | 1.10 | 3 | 1 | 5 | 0.92 | 0.92 | 0.68 | 0.65 |
| TR_ReschClin | 0.14 | 0.16 | 0.34 | 0.27 | 0.09 | 0.41 | 847 | 3.02 | 1.16 | 3 | 1 | 5 | 0.92 | 0.92 | 0.73 | 0.70 |
| TR_Patients | 0.12 | 0.15 | 0.36 | 0.28 | 0.09 | 0.41 | 846 | 3.05 | 1.13 | 3 | 1 | 5 | 0.91 | 0.91 | 0.74 | 0.71 |
| TR_Priorities | 0.13 | 0.15 | 0.41 | 0.24 | 0.07 | 0.41 | 845 | 2.97 | 1.10 | 3 | 1 | 5 | 0.92 | 0.92 | 0.70 | 0.67 |
| TR_PttOutcs | 0.09 | 0.09 | 0.34 | 0.35 | 0.13 | 0.41 | 845 | 3.33 | 1.10 | 3 | 1 | 5 | 0.91 | 0.91 | 0.76 | 0.72 |
| TR_OrgOutcs | 0.10 | 0.11 | 0.38 | 0.31 | 0.10 | 0.41 | 845 | 3.20 | 1.08 | 3 | 1 | 5 | 0.91 | 0.91 | 0.77 | 0.74 |
| TR_PrcChg | 0.11 | 0.12 | 0.39 | 0.29 | 0.09 | 0.41 | 845 | 3.14 | 1.09 | 3 | 1 | 5 | 0.91 | 0.91 | 0.77 | 0.74 |
| TR_Support | 0.12 | 0.13 | 0.40 | 0.25 | 0.10 | 0.41 | 845 | 3.09 | 1.13 | 3 | 1 | 5 | 0.92 | 0.92 | 0.69 | 0.66 |
| TR_Impact | 0.09 | 0.11 | 0.34 | 0.34 | 0.11 | 0.41 | 845 | 3.27 | 1.10 | 3 | 1 | 5 | 0.91 | 0.91 | 0.79 | 0.75 |
| TR_Consumers | 0.09 | 0.12 | 0.35 | 0.31 | 0.13 | 0.41 | 845 | 3.27 | 1.12 | 3 | 1 | 5 | 0.91 | 0.91 | 0.74 | 0.71 |
| Overall | | | | | | | | 31.17^c^ | 8.4 |  | | | 0.92 | 0.92 | 0.54 |  |
| ^a^Correlaiton of the item with the scale, adjusted for item overlap and scale reliability | | | | | | | | | | | | | | | | |
| ^b^Correlation of the item with the scale without the item in it | | | | | | | | | | | | | | | | |
| ^c^Mean of sum of items | | | | | | | | | | | | | | | | |

**Table S8: Research impact**: response frequencies, descriptive statistics, reliability and correlation

|  | Response Frequencies | | | | | | | Descriptive Statistics | | | | | Alpha | | Correlation | |
| --- | --- | --- | --- | --- | --- | --- | --- | --- | --- | --- | --- | --- | --- | --- | --- | --- |
| Variable | 1 | 2 | 3 | 4 | 5 | Missing | n | Mean | SD | Median | Min. | Max. | Raw | Std | Scale^a^ | Drop^b^ |
| RI_ClinChg | 0.05 | 0.07 | 0.29 | 0.46 | 0.13 | 0.43 | 817 | 3.56 | 0.98 | 4 | 1 | 5 | 0.91 | 0.91 | 0.71 | 0.68 |
| RI_Policy | 0.05 | 0.07 | 0.30 | 0.45 | 0.13 | 0.43 | 816 | 3.56 | 0.96 | 4 | 1 | 5 | 0.92 | 0.92 | 0.68 | 0.65 |
| RI_NewKnow | 0.05 | 0.06 | 0.27 | 0.47 | 0.16 | 0.43 | 817 | 3.63 | 0.98 | 4 | 1 | 5 | 0.91 | 0.91 | 0.74 | 0.70 |
| RI_Efficient | 0.06 | 0.09 | 0.36 | 0.38 | 0.11 | 0.43 | 817 | 3.38 | 1.00 | 3 | 1 | 5 | 0.91 | 0.91 | 0.75 | 0.72 |
| RI_CloseGap | 0.08 | 0.14 | 0.40 | 0.29 | 0.08 | 0.43 | 817 | 3.16 | 1.03 | 3 | 1 | 5 | 0.92 | 0.92 | 0.62 | 0.59 |
| RI_Economics | 0.07 | 0.11 | 0.45 | 0.31 | 0.06 | 0.43 | 817 | 3.18 | 0.94 | 3 | 1 | 5 | 0.92 | 0.92 | 0.64 | 0.62 |
| RI_PtOutcm | 0.05 | 0.06 | 0.28 | 0.48 | 0.13 | 0.43 | 817 | 3.57 | 0.98 | 4 | 1 | 5 | 0.91 | 0.91 | 0.76 | 0.73 |
| RI_SocOutcm | 0.07 | 0.11 | 0.38 | 0.36 | 0.09 | 0.43 | 817 | 3.30 | 1.01 | 3 | 1 | 5 | 0.91 | 0.91 | 0.73 | 0.70 |
| RI_PtExp | 0.06 | 0.08 | 0.32 | 0.42 | 0.11 | 0.43 | 817 | 3.45 | 1.00 | 4 | 1 | 5 | 0.91 | 0.91 | 0.73 | 0.70 |
| RI_Staff | 0.10 | 0.13 | 0.35 | 0.35 | 0.08 | 0.43 | 817 | 3.19 | 1.07 | 3 | 1 | 5 | 0.91 | 0.91 | 0.71 | 0.68 |
| RI_QualSafe | 0.06 | 0.06 | 0.30 | 0.45 | 0.13 | 0.43 | 817 | 3.54 | 0.98 | 4 | 1 | 5 | 0.91 | 0.91 | 0.75 | 0.72 |
| RI_Enviro | 0.09 | 0.13 | 0.42 | 0.28 | 0.07 | 0.43 | 817 | 3.11 | 1.04 | 3 | 1 | 5 | 0.92 | 0.92 | 0.61 | 0.59 |
| Overall | | | | | | | | 40.34^c^ | 8.88 |  | | | 0.89 | 0.92 | 0.49 |  |
| ^a^Correlation with the scale, adjusted for item overlap and scale reliability | | | | | | | | | | | | | | | | |
| ^b^Correlation with the scale without the item in it | | | | | | | | | | | | | | | | |
| ^c^Mean of sum of items | | | | | | | | | | | | | | | | |

**Table S9: Individual Research Capability:** response frequencies, descriptive statistics, reliability and correlation

|  | Response Frequencies | | | | Descriptive Statistics | | | | | Alpha | | Correlation | |
| --- | --- | --- | --- | --- | --- | --- | --- | --- | --- | --- | --- | --- | --- |
| Variable | No | Yes | Missing | n | Mean | SD | Median | Min. | Max. | Raw | Std | Scale^a^ | Drop^b^ |
| CapY_Prop | 0.90 | 0.10 | 0 | 1,430 | 0.10 | 0.30 | 0 | 0 | 1 | 0.88 | 0.89 | 0.71 | 0.65 |
| CapY_Litsch | 0.71 | 0.29 | 0 | 1,430 | 0.29 | 0.45 | 0 | 0 | 1 | 0.88 | 0.89 | 0.67 | 0.65 |
| CapY_Fund | 0.93 | 0.07 | 0 | 1,430 | 0.07 | 0.25 | 0 | 0 | 1 | 0.89 | 0.90 | 0.47 | 0.44 |
| CapY_Consum | 0.73 | 0.27 | 0 | 1,430 | 0.27 | 0.44 | 0 | 0 | 1 | 0.89 | 0.89 | 0.58 | 0.56 |
| CapY_Stakeh | 0.82 | 0.18 | 0 | 1,430 | 0.18 | 0.39 | 0 | 0 | 1 | 0.88 | 0.89 | 0.63 | 0.61 |
| CapY_Ethics | 0.91 | 0.09 | 0 | 1,430 | 0.09 | 0.29 | 0 | 0 | 1 | 0.88 | 0.89 | 0.65 | 0.59 |
| CapY_Data | 0.68 | 0.32 | 0 | 1,430 | 0.32 | 0.47 | 0 | 0 | 1 | 0.88 | 0.89 | 0.69 | 0.67 |
| CapY_Datman | 0.82 | 0.18 | 0 | 1,430 | 0.18 | 0.39 | 0 | 0 | 1 | 0.88 | 0.88 | 0.75 | 0.71 |
| CapY_Stat | 0.93 | 0.07 | 0 | 1,430 | 0.07 | 0.25 | 0 | 0 | 1 | 0.89 | 0.89 | 0.56 | 0.51 |
| CapY_Qual | 0.90 | 0.10 | 0 | 1,430 | 0.10 | 0.30 | 0 | 0 | 1 | 0.88 | 0.89 | 0.68 | 0.62 |
| CapY_Report | 0.87 | 0.13 | 0 | 1,430 | 0.13 | 0.33 | 0 | 0 | 1 | 0.88 | 0.88 | 0.75 | 0.68 |
| CapY_Public | 0.91 | 0.09 | 0 | 1,430 | 0.09 | 0.29 | 0 | 0 | 1 | 0.88 | 0.89 | 0.70 | 0.63 |
| Overall | | | | | 1.88^c^ | 2.87 |  | | | 0.89 | 0.9 | 0.42 |  |
| ^a^Correlation with the scale, adjusted for item overlap and scale reliability | | | | | | | | | | | | | |
| ^b^Correlation with the scale without the item in it | | | | | | | | | | | | | |
| ^c^Mean of sum of items | | | | | | | | | | | | | |

**Table S10: Team Research Capability:**  response frequencies, descriptive statistics, reliability and correlation

|  | Response Frequencies | | | | Descriptive Statistics | | | | | Alpha | | Correlation | |
| --- | --- | --- | --- | --- | --- | --- | --- | --- | --- | --- | --- | --- | --- |
| Variable | No | Yes | Missing | n | Mean | SD | Median | Min. | Max. | Raw | Std | Scale^a^ | Drop^b^ |
| CapAborT | 0.81 | 0.19 | 0 | 1,430 | 0.19 | 0.40 | 0 | 0 | 1 | 0.96 | 0.96 | 0.77 | 0.76 |
| CapConsumT | 0.80 | 0.20 | 0 | 1,430 | 0.20 | 0.40 | 0 | 0 | 1 | 0.97 | 0.97 | 0.68 | 0.66 |
| CapDataT | 0.78 | 0.22 | 0 | 1,430 | 0.22 | 0.41 | 0 | 0 | 1 | 0.97 | 0.97 | 0.68 | 0.66 |
| CapDatmanT | 0.79 | 0.21 | 0 | 1,430 | 0.21 | 0.41 | 0 | 0 | 1 | 0.96 | 0.96 | 0.83 | 0.81 |
| CapEconT | 0.82 | 0.18 | 0 | 1,430 | 0.18 | 0.38 | 0 | 0 | 1 | 0.96 | 0.96 | 0.86 | 0.84 |
| CapEthicsT | 0.80 | 0.20 | 0 | 1,430 | 0.20 | 0.40 | 0 | 0 | 1 | 0.96 | 0.96 | 0.88 | 0.86 |
| CapFundT | 0.78 | 0.22 | 0 | 1,430 | 0.22 | 0.42 | 0 | 0 | 1 | 0.96 | 0.96 | 0.84 | 0.83 |
| CapLitschT | 0.82 | 0.18 | 0 | 1,430 | 0.18 | 0.39 | 0 | 0 | 1 | 0.96 | 0.97 | 0.72 | 0.71 |
| CapPropT | 0.78 | 0.22 | 0 | 1,430 | 0.22 | 0.41 | 0 | 0 | 1 | 0.96 | 0.96 | 0.85 | 0.84 |
| CapPublicT | 0.80 | 0.20 | 0 | 1,430 | 0.20 | 0.40 | 0 | 0 | 1 | 0.96 | 0.96 | 0.90 | 0.88 |
| CapQualT | 0.81 | 0.19 | 0 | 1,430 | 0.19 | 0.39 | 0 | 0 | 1 | 0.96 | 0.96 | 0.89 | 0.86 |
| CapReportT | 0.80 | 0.20 | 0 | 1,430 | 0.20 | 0.40 | 0 | 0 | 1 | 0.96 | 0.96 | 0.89 | 0.87 |
| CapStakehT | 0.79 | 0.21 | 0 | 1,430 | 0.21 | 0.40 | 0 | 0 | 1 | 0.96 | 0.96 | 0.81 | 0.79 |
| CapStatT | 0.80 | 0.20 | 0 | 1,430 | 0.20 | 0.40 | 0 | 0 | 1 | 0.96 | 0.96 | 0.89 | 0.87 |
| Overall | | | | | 2.82^c^ | 4.67 |  | | | 0.97 | 0.97 | 0.67 |  |
| ^a^Correlation with scale, adjusted for item overlap and scale reliability | | | | | | | | | | | | | |
| ^b^Correlation with the scale without the item in it | | | | | | | | | | | | | |
| ^c^Mean of sum of items | | | | | | | | | | | | | |
