# Supplementary material for: The development and validation of the Research for Practice Tool (R4PT) for nursing and midwifery
Source: BMC Health Serv Res. 2025 Sep 30;25:1245. doi: 10.1186/s12913-025-13112-x (PMC12482206; doi:10.1186/s12913-025-13112-x)
Supplement: Supplementary file 3 — Supplementary Material 3. [file 12913_2025_13112_MOESM3_ESM.docx]

Database: Medline <1946 - present>

Search Strategy:

--------------------------------------------------------------------------------

1 exp nurses/ or nursing staff/ (114486)

2 (nurs* or midwif* or midwives).ti,ab,kw,kf. (516329)

3 1 or 2 (555964)

4 Capacity building/ (3148)

5 (capacit* adj2 (build* or develop*)).ti,ab,kw,kf. (13101)

6 Research Personnel/ (18467)

7 exp Research/ (671741)

8 Research support as topic/ (22876)

9 Health Services Research/ (38040)

10 exp Nursing Research/ (53179)

11 Translational medical research/ (12257)

12 (research* adj3 (engag* or enhanc* or uptak* or up-tak* or enact* or capacit* or capabil* or participat* or involv* or culture or activ* or strateg* or impact*)).ti,ab,kw,kf. (82153)

13 4 or 5 (14394)

14 6 or 7 or 8 or 9 or 10 or 11 or 12 (749220)

15 3 and 13 and 14 (364)

Database: Embase <1996 to current>

Search Strategy:

--------------------------------------------------------------------------------

1 exp nurse/ (168692)

2 nursing staff/ (54969)

3 (nurs* or midwif* or midwives).ti,ab,kw,kf. (459557)

4 1 or 2 or 3 (508786)

5 capacity building/ (6031)

6 (capacit* adj2 (build* or develop*)).ti,ab,kw,kf. (15416)

7 5 or 6 (17098)

8 personnel/ (15361)

9 exp research/ (793163)

10 health services research/ (29276)

11 (research* adj3 (engag* or enhance* or uptak* or up-tak* or enact* or capacit* or participat* or involv* or culture or activ* or strateg* or impact*)).ti,ab,kw,kf. (94520)

12 8 or 9 or 10 or 11 (893285)

13 4 and 7 and 12 (453)

Web of Science

**1 (nurs* OR midwif* OR midwives)** (Topic) 374,795

**2 (capacit* Near/2 (build* or develop*))** (Topic) 35,558

**3 (research* Near/3 (engag* or enhanc* or uptak* or up-tak* or enact* or capacit* or capabil* or participat* or involv* or culture or activ* or strateg* or impact*))** (Topic) 239,612

1 AND 2 AND 3 204

Proquest Health and Medicine

noft((nurs* or midwif* or midwives)) AND noft((capacity N/2 (build* or develop))) AND noft((research* N/3 (engag* or enhanc* or uptak* or up-tak* or enact* or capacit* or capabil* or participat* or involv* or culture or activ* or strateg* or impact*))) limited to Scholarly journals an English language publications 186
